# Blockchain-Based Healthcare Credentialing: A Solution to High Costs and Administrative Burdens

**DOI:** 10.7759/cureus.63318

**Published:** 2024-06-27

**Authors:** Ebenezer Chinedu-Eneh, Priya Ramaswamy, Patrick E Farmer

**Affiliations:** 1 Anesthesiology and Perioperative Care, School of Medicine, University of California, San Francisco, San Francisco, USA; 2 Anesthesia and Perioperative Care, University of California, San Francisco, San Francisco Veterans Affairs Medical Center, San Francisco, USA; 3 Anesthesiology, University of California San Diego, San Diego, USA

**Keywords:** medical staffing, labor costs, delivery of healthcare, patient outcomes, credentialing, blockchain in medicine

## Abstract

This article proposes a blockchain-based system to address the inefficiencies of the current healthcare credentialing process that contribute to workforce shortages. Leveraging blockchain's unique features, the proposed system aims to reduce time, cost, and labor, offering significant time savings, increased trustworthiness, and enhanced staffing resilience. Real-world blockchain examples demonstrate the feasibility of this approach. The study concludes that a blockchain-based credentialing system could streamline healthcare credentialing, enhance preparedness for future challenges, and improve healthcare delivery and patient outcomes.

## Editorial

The healthcare sector is currently grappling with an inefficient credentialing system, which is not only labor-intensive but also hinders the system's capacity to address staff shortages and make accurate workforce projections swiftly. The COVID-19 pandemic further underscored this issue, with many states having to temporarily suspend their extensive credentialing and licensing processes to cope with the crisis [1,2]. However, with the suspensions being only temporary, the disorganization has returned and the waste costs Americans upwards of $15 billion per year [3].

The inefficiencies in the current system extend beyond financial implications, significantly impacting patient care by delaying the integration of qualified healthcare professionals into the workforce. This delay exacerbates staff shortages, leading to longer patient wait times and increased workloads for current staff, which can compromise the quality of care delivered [4]. For healthcare professionals, these inefficiencies mean prolonged periods of uncertainty, potential loss of income, and the frustration of navigating a cumbersome system, which can deter talented individuals from entering or remaining in the healthcare field [5]. Overall, the inefficiency of the healthcare credentialing system undermines the effectiveness and sustainability of healthcare delivery, making it imperative to seek more streamlined and efficient solutions.

**Figure 1:**
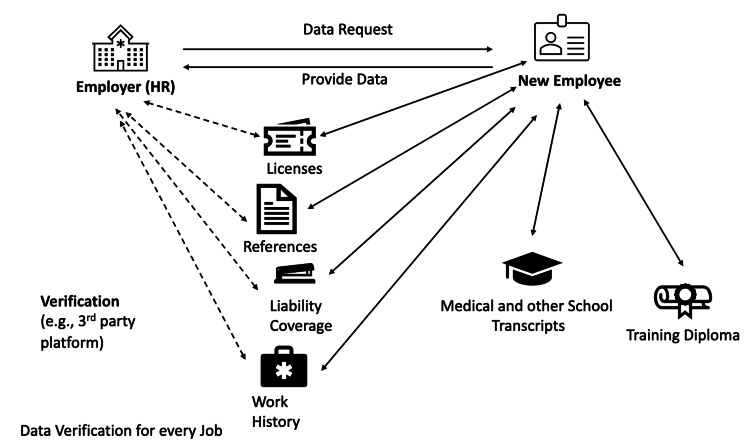
Schematic diagram detailing the interactive data verification process in health professional employment. It represents new employees and employers as nodes, interconnected by two-way arrows to various data sources, such as licensure agencies, referees, liability coverage providers, and educational institutions. These arrows signify the dynamic exchange of data — tracking, sending, and receiving — required from the employee and the employer to each source. This intricate network underscores the complexity and potential inefficiency of the current verification process. Courtesy: Authors.

The current credentialing process within the medical field is fraught with inefficiencies which contributes to workforce shortage for anesthesiologists and other physicians. Periodic re-credentialing when one changes employers involves re-verifying many of the same documents required for state licensure, and these elements are often subject to change. Each entity, such as hospitals and insurance plans, must independently verify every credential [1]. As illustrated in Figure 1, this verification means that whenever a candidate is hired, all parties involved must individually contact the candidate's previous educational institutions and employers to confirm qualifications, certifications, and work experience [1,3].

**Figure 2:**
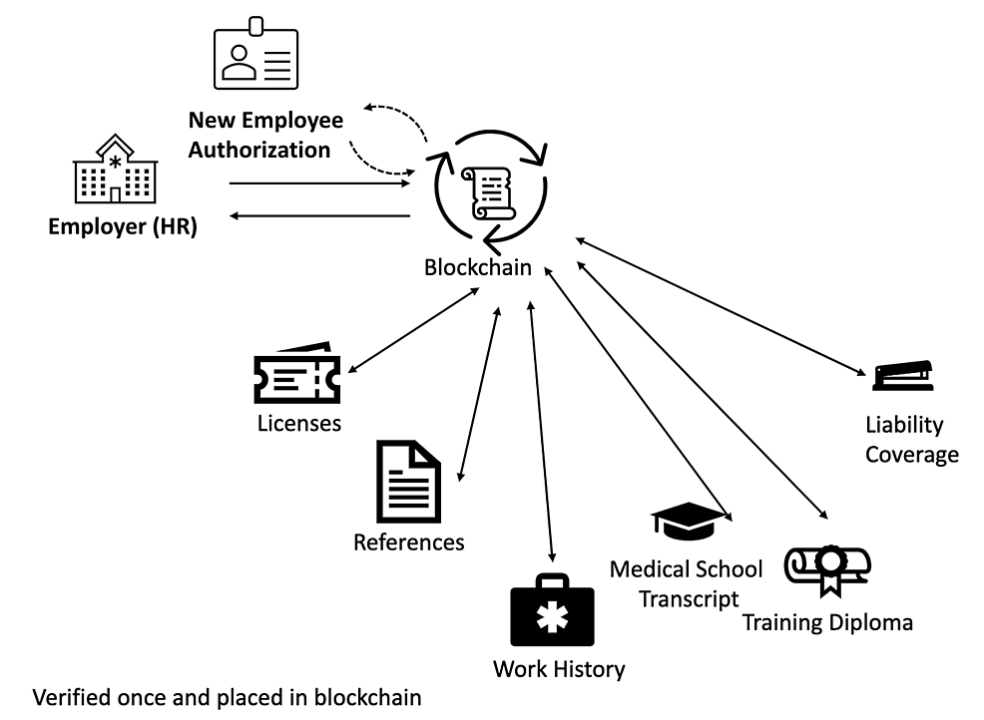
Revolutionizing data verification workflow in healthcare: a blockchain approach. The figure showcases a data verification workflow for healthcare professionals, utilizing blockchain technology as a secure, trusted repository of critical employment data. Two-way arrows demonstrate the secure and verified flow of information, such as licenses, references, work history, and transcripts, from the respective sources onto the blockchain, highlighting the system's user-friendliness for these stakeholders. The authorization arrow from the employee icon represents the employee's ability to manage their data on this blockchain repository, including adding and removing items as necessary. The cyclic arrows around the blockchain script icon signify the automation of the blockchain, running smart contracts to ensure data validity and integrity. Furthermore, two-way arrows from the blockchain to both the employer and employee icons illustrate the system's bilateral accessibility, simplifying the process and fostering transparency. Courtesy: Authors

We are at a point where our systems can become more intelligently organized and remove waste in the process. Accomplishing this with blockchain technology offers several key advantages that make it an ideal solution for enhancing the safety and efficiency of healthcare credentialing. As illustrated in Figure 2, transforming credential verification into a dynamic, automated, and instantaneous system offers substantial advantages to all key stakeholders.

This paper highlights three persuasive reasons to consider adopting blockchain for credentialing purposes: significant time savings, increased trustworthiness, and enhanced staffing resilience. We will explore how blockchain technology can significantly improve healthcare administration efficiency and reduce its financial burden, eliminate redundancies while balancing the tension between security and transparency, and strengthen the healthcare sector by increasing resiliency within the workforce.

Significant time savings

The United States healthcare system, which spends over $300 billion annually on administrative costs, has the potential to save up to $29,000 per physician through reforms [3]. Reducing these costs is the duty of the healthcare system, and it will likely take many reforms that cumulatively add up to allay this expenditure. One area for improvement is physician credentialing.

The task of verifying a physician's qualifications and certifications takes an average of 120 days [1] contributes significantly to these costs. Yet, the emergence of credentialing companies has not provided an effective solution, as evidenced by a 2018 Centers for Medicare and Medicaid Services audit which revealed nearly half of provider directory locations had at least one inaccuracy [6]. A more efficient solution is needed, and blockchain technology presents a compelling case.

Consider the transformation blockchain had on the traditional remittance system. Traditionally, this vital process of sending money overseas to support family members typically took about one to five business days to complete. Furthermore, a 2019 visa report revealed that approximately 200 million workers sent $706 billion in global remittances, incurring fees of 7% and 6.66% through conventional banking and cash remittance methods, resulting in nearly $50 billion in fees [7].

In contrast, the Bitcoin blockchain system, which holds over $400 billion in value, processes more than 12,000 transactions per hour, with most transactions completed in 10 minutes [8]. These transactions have a median fee of 0.34%, a stark contrast to the traditional remittance system.

Similarly, the Ethereum platform offers a glimpse into the potential of Smart Contracts, with over 48,000 transactions per hour, a mean transaction fee of 0.16%, and an average completion time of eight minutes [9]. Smart Contracts are software programs that automatically execute when contractual terms are met, suggesting a future where healthcare credentialing could occur outside working hours, effectively reducing the associated costs and time to almost zero. By leveraging blockchain technology, we can significantly improve healthcare administration efficiency and reduce its financial burden.

Increased trustworthiness

In addition to time and cost savings, a blockchain credentialing system eliminates redundancies and balances the tension between security and transparency. Currently, stakeholders must individually contact a candidate's prior educational institutions and employers to verify qualifications, certifications, and work history [1]. This tedious process can be transformed with the implementation of blockchain technology.

Imagine bridging current communication gaps via a smart network where employers, employees, and credentialing bodies are interconnected. Within this network, credentialing organizations could interact directly with the blockchain, importing verified provider credentials through Smart Contracts. This method would ensure an accurate and immutable representation of a health professional's credentialing history.

Consider the potential for individuals to share their profiles with prospective employers, using private passwords, or “private keys”, to maintain control over what information they share. Smart Contracts could then assess whether a health professional's credentials align with the employer's requirements, making connections accordingly. As illustrated in Figure 2, automatic renewal functions could also be incorporated to notify employees and employers when credentials are nearing expiration and initiate the renewal process. The transparency of publicly available smart contract mechanics also reduces the risk of party bias or error.

The potential for implementing a secure credentialing blockchain system is vast, with various avenues for execution. These include deployment through the credential-issuing institution, a comprehensive government system that encompasses all physician credentials, or a third party creating a blockchain for institutions and individuals to utilize.

Evidence of successful implementations can be seen in Ethiopia, Estonia, and Georgia. Per IOHK, the Ethiopian education sector has embraced blockchain technology by creating tamper-proof records for 3,500 schools, five-million students, and 750,000 teachers [10]. This innovation has streamlined the management of educational records, a critical component for employment opportunities. Georgia has also made strides in this space, becoming the first country to add graduating seniors' certificates to the blockchain. This move showcases the trust and scalability of the system [11].

Estonia, on the other hand, has leveraged blockchain technology to enhance its healthcare sector. Information on all medical treatments performed can now be retrieved by healthcare professionals or health insurance companies using the Guardtime Blockchain [12].

These examples demonstrate the potential of blockchain technology to revolutionize vital public infrastructures, including healthcare. The flexibility and adaptability of blockchain technology allow institutions to collaborate, creating interconnected blocks for credentialing purposes, and tailoring the system to suit various contexts.

Enhanced staffing resilience

This interconnectedness strengthens the healthcare sector by increasing resiliency within the workforce. In recent times, there has been a shift in the labor-market dynamics between medical institutions and healthcare professionals, with healthcare professionals increasingly prioritizing organizations that share their values. Given the time savings and increased trustworthiness a blockchain credentialing system provides, hospitals can leverage blockchain technology to attract healthcare workers and improve their job satisfaction, which play a significant role in their employment choices. It's vital for healthcare professionals to have a positive work environment as it fosters a sense of fulfillment and draws like-minded individuals.

By implementing a dependable and user-friendly blockchain system, hospitals can streamline the hiring process, resulting in greater efficiency and increased staffing. This approach may even encourage healthcare professionals who have left the field to return, therefore ultimately enhancing the quality of care, patient outcomes, and overall satisfaction.

Addressing concerns and skepticism

Understandably, integrating blockchain technology in healthcare raises concerns regarding data privacy and security. Skepticism within the industry is not unfounded, given the novelty of the technology and its association with high-profile cyber incidents. However, blockchain's inherent design-decentralization, cryptographic hashing, and consensus mechanisms-provides a robust framework for secure data management [13]. Decentralization reduces the risk of a single point of failure; cryptographic hashing ensures data integrity and consensus mechanisms validate transactions [14]. Counterarguments to skepticism include the technology's track record in sectors such as finance and supply chain management, where it has enhanced transparency and security [15]. Moreover, regulatory compliance and advancements in blockchain technology continue to address these concerns, making it a viable solution for improving healthcare administration while safeguarding sensitive information [16].

In conclusion, the future of efficient healthcare credentialing lies in the adoption of blockchain-based systems. The potential for rapid, secure, and effective solutions to the current challenges is immense. Moreover, the true transformative power of this technology could be realized if medical boards, such as the American Board of Medical Specialties or the American Board of Anesthesiology, take the lead in spearheading these advancements. By doing so, they can ensure that the solutions developed are tailored to the unique needs and challenges of the healthcare sector.

The time for lamenting the lengthy and cumbersome credentialing process is over. The conversation must now shift towards the tangible benefits of adopting a blockchain credentialing system - a system that promises rapidity, efficiency, and reliability. As we look towards the future of healthcare, blockchain technology holds the key to unlocking unprecedented levels of efficiency and trust in the credentialing process.
